# Human Milk Endocannabinoid Levels as a Function of Obesity and Diurnal Rhythm

**DOI:** 10.3390/nu13072297

**Published:** 2021-07-03

**Authors:** Palika Datta, Michael W. Melkus, Kathleen Rewers-Felkins, Dhavalkumar Patel, Tiffany Bateman, Teresa Baker, Thomas W. Hale

**Affiliations:** 1 Department of Pediatrics, School of Medicine, Texas Tech University Health Sciences Center, Amarillo, TX 79106, USA; Palika.D.Datta@ttuhsc.edu (P.D.); Kathleen.Felkins@ttuhsc.edu (K.R.-F.); 2 InfantRisk Center of Excellence, School of Medicine, Texas Tech University Health Sciences Center, Amarillo, TX 79106, USA; Teresa.Baker@ttuhsc.edu; 3 Department of Surgery, School of Medicine, Texas Tech University Health Sciences Center, Lubbock, TX 79430, USA; Michael.Melkus@ttuhsc.edu; 4 Department of Pharmaceutical Sciences, Jerry H. Hodge School of Pharmacy, Texas Tech University Health Sciences Center, Amarillo, TX 79106, USA; Dhavalkumar.Patel@ttuhsc.edu; 5 Baptist St. Anthony’s Hospital, Amarillo, TX 79106, USA; outstandingtiffany@yahoo.com; 6 Department of Obstetrics and Gynecology, School of Medicine, Texas Tech University Health Sciences Center, Amarillo, TX 79106, USA

**Keywords:** endocannabinoids, human milk, 2-arachidonoylglycerol, anandamide, obesity, diurnal

## Abstract

The endocannabinoid system is involved in the regulation of a variety of physiological and cognitive processes. While the endocannabinoids 2-arachidonoylglycerol (2-AG) and anandamide (*N*-arachidonoylethanolamine, AEA) have been found in breast milk, their role(s) have yet to be determined. This study determined the normal concentration ranges of endocannabinoids (2-AG and AEA) in breast milk and the influences, if any, of obesity and diurnal rhythms on their levels. Milk samples were collected from 36 breastfeeding mothers at 4–8 weeks postpartum at each feed over a 24-h period, and further stratified into three groups based on body mass index (BMI). The samples were analyzed using liquid chromatography mass spectrometry. AEA was below the limit of detection and 2-AG levels averaged 59.3 ± 18.3 ng/mL (± SD) in women with normal BMI. Wide-ranging 2-AG concentrations in the overweight (65.5 ± 41.9 ng/mL) /obese (66.1 ± 40.6 ng/mL) groups suggest BMI may be a contributing factor influencing its levels. Following a diurnal pattern, there was a significantly higher 2-AG concentration observed during the day, as compared to night time samples. In conclusion, our study clearly suggests that appropriate milk collection and storage conditions are critical. Further, body weight and diurnal rhythm appear to influence levels of 2-AG. Based on these results, future studies are underway to determine what specific roles endocannabinoids may play in human milk and how elevated levels of 2-AG may modulate infant appetite and health.

## 1. Introduction

Breastfeeding is universally recommended as the best choice of infant feeding for at least the first 6–12 months [1]. Nature created breastmilk to provide not only optimal nutrition, but to also support adequate growth and maturation during infancy. Breast milk is rich in lipids and fatty acids and provides almost all energy requirements for neonates up to 6 months of age [2]. Arachidonic acid (ARA) is the most predominant long-chain polyunsaturated fatty acid in human milk and is essential for infant development [3]. Human milk ARA levels are modulated by dietary intake and vary depending on dietary habits among mothers. ARA serves as a precursor to eicosanoids and endocannabinoids that are also found in human milk [4]. The endocannabinoid system (ECS) has been implicated in the regulation of a variety of physiological and cognitive processes including fertility [5], pregnancy [6], both pre- and postnatal development [7], appetite [8], pain-perception [9] and in mediating the pharmacological effects of cannabis [10].

The psychoactive constituent in cannabis, Δ^9^-tetrahydrocannabinol (THC), was isolated in the mid-1960s. Determining how this phytocannabinoid worked eventually led to the discovery of the endocannabinoid receptors, CB1 and CB2 [11], including the major endogenous cannabinoids (anandamide and 2-arachidonoyl glycerol) 20 years later. Endocannabinoids are lipid-based molecules that also bind to and activate endocannabinoid receptors CB1 and CB2. They are referred to as retrograde inhibitors in that they apparently suppress parasympathetic synapses. Anandamide (AEA) is a fatty acid neurotransmitter derived from the non-oxidative metabolism of eicosatetraenoic acid (arachidonic acid) [12] 2-AG is synthesized from arachidonic acid-containing diacylglycerol (DAG), which is derived from the increase in inositol phospholipid metabolism by the action of diacylglycerol lipase [13]. Unlike anandamide, the formation of 2-AG is calcium-dependent and is mediated by the activities of phospholipase C (PLC) and diacylglycerol lipase (DAGL).

The three key components of the ECS are small molecular weight endocannabinoids that activate cannabinoid receptors, and the metabolic enzymes that break down endocannabinoids after they bind to their targets. While the endocannabinoids were previously found in human milk, nothing has yet been published defining their function in human milk. It has been suggested they may initiate infant suckling [7], they may stimulate infant appetite and a feeling of well-being, and may also play a role in infant brain and neuronal development [14,15]. However, at present there is virtually no understanding of the roles of endocannabinoids in human milk. Unfortunately, several previous studies have unintentionally introduced errors in determining endocannabinoid levels due to the collection and storage process before analyzing the samples [4,16]. It was previously demonstrated that milk stored at 4 degrees undergoes a metabolic process that results in higher levels of 2-AG compared to the initial collection concentrations [16]. The purpose of our study was to determine the normal concentration ranges of endocannabinoids (2-AG and AEA) in human milk using strict collection and storage conditions. In addition, we sought to determine the influence of obesity, circadian\diurnal rhythm and longitudinal breastfeeding effects on endocannabinoid levels in human milk.

## 2. Materials and Methods

### 2.1. Study Design

This research study was designed to explore the effects of obesity, diurnal rhythm and longitudinal breastfeeding on endocannabinoid levels in breastmilk. However, initial experiments were first conducted to accurately determine endocannabinoid concentrations in breast milk and validate appropriate collection/storage conditions. These experiments found that if left at room temperature or even −20 °C, levels of 2-AG increased within hours. Because of the variable levels found in milk as a function of time, milk samples were subsequently collected (n = 6) and analyzed on the same day (fresh) and individual groups stored at different temperature conditions (4 °C, −20 °C and −80 °C) for 24 h, 1 week and 1 month.

In our subsequent study, milk samples were collected from breastfeeding mothers and stratified into 3 groups based on their BMI calculated at the time of the study. They were termed as normal weight group (BMI ≤ 25) [n = 12], overweight group (25 ≤ BMI ≤ 30) [n = 11] and obese group (BMI ≥ 30) [n = 13] according to Centers for Disease Control guidelines [17]. Milk samples were collected during regular feeding times for 24 h and analyzed for AEA and 2-AG concentration for each group. To determine the effect of diurnal rhythm, collected milk samples were divided into day samples (6 am–10 pm) and night samples (11 pm–5 am) and analyzed. One participant in the normal BMI group was excluded since she did not provide any night samples. For the longitudinal study, women provided milk samples over a 24-h period again at 4–6 months for analysis. Due to a high attrition rate during the study, we only analyzed 4 women for each of the 3 BMI groups. 

### 2.2. Study Participants

The inclusion criteria for our study included mothers who were exclusively breastfeeding, at least 37 weeks of gestation and between 4-8 weeks post-partum. The exclusion criteria included women who were pregnant, had had any form of breast surgery/augmentation, and were consuming any medications including antiviral, corticosteroids, antidepressants or pain killers (including aspirin, acetaminophen, ibuprofen, etc.). This study was approved by the Institutional Review Board (A19-4070). All the subjects had informed consent signed prior to the collection of milk samples.

### 2.3. Subject Recruitment and Collection

Potential subjects were recruited by a local board-certified lactation consultant. Women who met the inclusion criteria and volunteered to participate were then consented. Previously we analyzed both fore and hind milk and found no significant difference in endocannabinoid levels (data not shown), therefore samples for analysis were collected as a whole fraction. Breast milk samples were collected under the subject's normal breastfeeding conditions and their usual feeding pattern for a 24-h period in order to prevent altering their natural endocannabinoid levels. After feeding their child, the subject was asked to pump both their breasts simultaneously, gently mix the milk from both breasts, and pour the sample into a collection tube provided. The tubes were labelled with the time of collection and immediately stored in dry ice. These samples were subsequently collected by research personnel at 24 h, placed in a −80 °C freezer and analyzed within 1 week of collection.

### 2.4. Quantitation Approach and Sample Analysis

The optimal chromatographic conditions were selected for the resolution and sensitivity for the analytes AEA and 2-AG. These analytes are present endogenously, therefore obtaining a blank matrix (analyte-free milk sample) was very challenging. A surrogate analyte approach was found to be a good solution. A surrogate analyte is defined as a molecule which shares similar physico-chemical properties, has identical chromatographic retention and recovery properties to the original analyte. The calibration curve was generated using deuterated standards, which served as surrogate analytes AEA-d8 and 2-AG-d5 in a true biological matrix (milk sample). In addition, these deuterated forms also served as internal standards (IS) for each other. The response factor (RF) was determined by analysis of AEA and AEA-d8, 2-AG and 2-AG-d5 prepared in acetonitrile each time the experiment was performed. By employing a surrogate approach, calibration curves were generated via adding a constant concentration of IS (50 ng/mL) and increasing the amount of each surrogate analyte into human milk. The peak areas of each surrogate analyte were detected and correlated with IS to determine the regression equation and regression coefficient of these calibration curves.

The analyte and standards were analyzed by AB Sciex QTRAP 5500 ultrahigh-performance liquid chromatography tandem mass spectrometry in the positive ion mode. Chromatography separation was performed on a Phenomenex Luna C-18 column, 50 × 2 mm, 3-micron particle size with a gradient elution. The flow rate was 0.5 mL/min. The autosampler was kept at 4 °C to prevent analyte degradation. The identification and quantification of analytes were confirmed by comparison of precursor and production m/z values and retention times with standards. Milk sample extraction was accomplished using protein precipitation. Surrogate deuterated analyte and internal standard were used to determine the calibration curve for each analyte and concentrations (ng/mL) of breast milk were calculated. Each sample was analyzed in triplicate and concentrations were averaged.

### 2.5. Statistical Analysis

Statistical analyses were performed using GraphPad Prism, version 9.1.0. Descriptive statistics (mean, standard deviation, standard error of the mean and range) were used for numeric variables. We used one-way ANOVA for continuous variable and Fisher’s Exact Test for categorical variable. Comparisons between weight groups for 2-AG concentrations were performed using unpaired, 2 tail, Student’s *t*-test. For diurnal (Day vs. Night) and longitudinal (4–8 Weeks vs. 4–6 Months) comparisons within each group were performed using a paired, 2-tail, Student’s *t*-test. Differences were considered statistically significant with a *p* ≤ 0.05.

## 3. Results

### 3.1. Milk Collection and Storage Conditions Affect Endocannabinoid Levels

**Figure 1:**
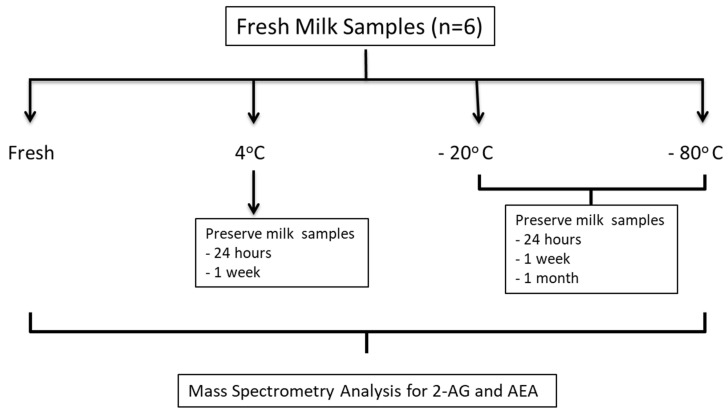
Schematic for milk sample collection, storage conditions and analysis. Fresh milk samples were collected and analyzed for 2-AG and AEA. The samples were aliquoted and stored under different conditions to determine how temperature (4 °C, −20 °C and −80 °C) and length of time (24 h, 1week and 1 month) affects endocannabinoid concentrations.

Our initial experiment was to determine the best collection and storage conditions for the analysis of endocannabinoids in breast milk (Figure 1).

**Figure 2:**
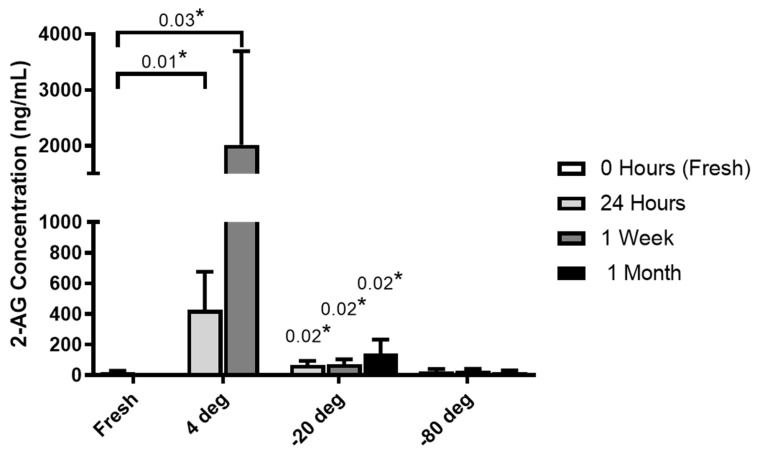
Endocannabinoid 2-AG concentrations for different storage conditions. Milk samples from 6 donors were analyzed on the same day (fresh) and stored at different temperature conditions (4 °C, −20 °C and −80 °C) for 24 h, 1 week and 1 month for 2-AG and AEA by ultrahigh-performance liquid chromatography tandem mass spectrometry. Each condition was analyzed using a paired Student’s *t* test with * *p* ≤ 0.05 being statistically significant.

Milk samples from each donor (n = 6) were collected and analyzed for AEA and 2-AG, which demonstrated that the method and length of time for the storage of breast milk samples significantly changes the level of endocannabinoids during storage. The storage of milk at 4 °C and −20 °C produced a significant increase in 2-AG levels, whereas AEA was not detected under any of the conditions. No substantial increase in 2-AG levels was noted in samples stored at −80 °C (Figure 2) over a period of 1 month.

### 3.2. Comparison of Milk Endocannabinoid Levels for Normal versus Obese Women

**Figure 3:**
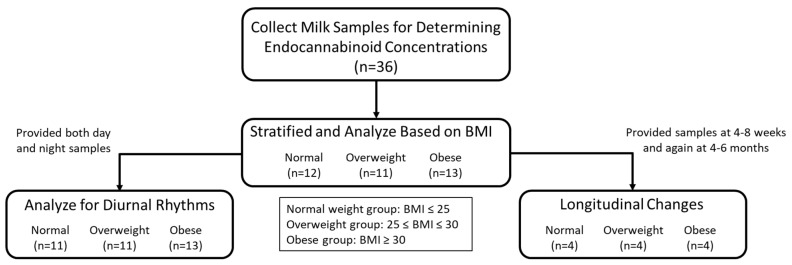
Flow chart for experimental design. N values shown for number of participants that met experimental criteria.

A total of 36 women consented to the study (Figure 3). Table 1 describes the demographics for maternal and infant characteristics.

**Figure 4:**
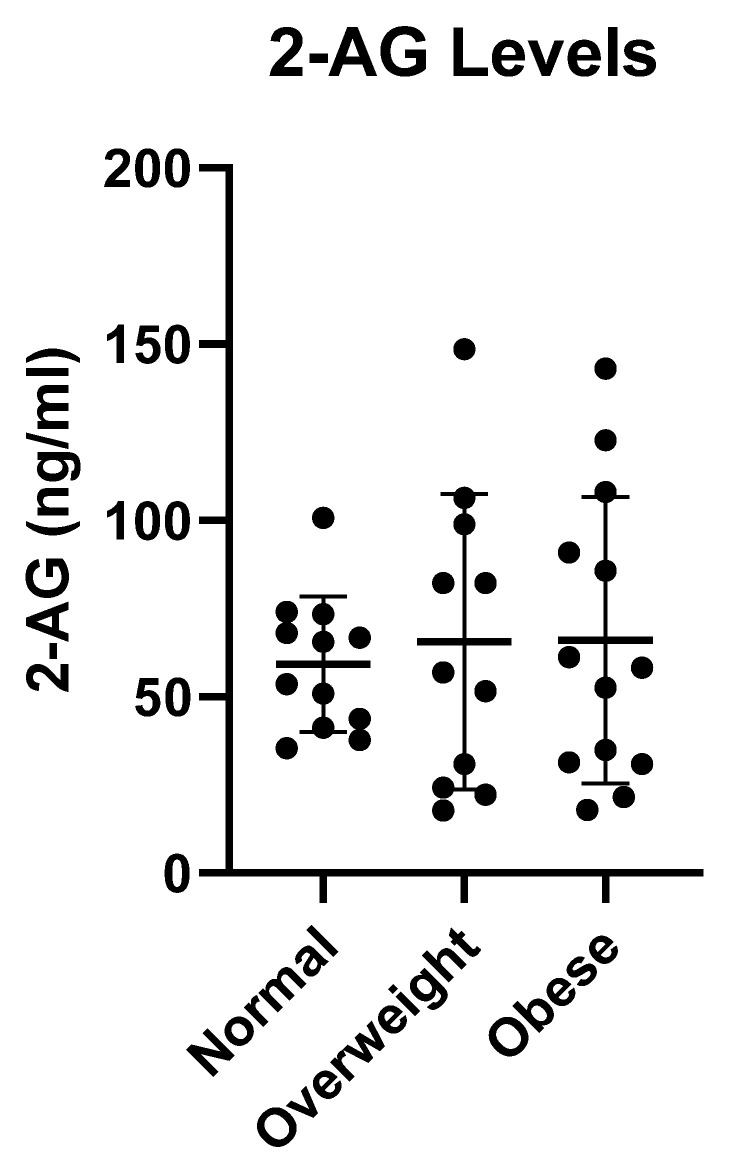
Concentration of 2-AG in breast milk for different body mass index (BMI) groups. Volunteer participants were stratified into 3 weight groups based on their BMI: (normal weight group (BMI ≤ 25) [n = 12], overweight group (25 ≤ BMI ≤ 30) [n = 11] and obese group (BMI ≥ 30) [n = 13]. Milk samples were collected over a 24 h period and the average 2-AG concentration determined.

All women participating in the study were between 21 and 39 years of age. Their BMI ranged from 19.6 to 51.6 kg/m^2^ and on an average they fed their child approximately nine times in a 24-h period of collection. No participants reported any food restrictions such as vegetarian or vegan diets. In a dataset of 263 milk samples collected for this study, 2-AG concentration levels were quantified and ranged from 13.1 to 221 ng/mL. The average levels of 2-AG were compared among the three BMI weight groups with no significant difference determined (Figure 4), which would imply overweight/obesity did not directly alter 2-AG breast milk levels compared to normal weight women. However, observing the average data for individuals, the normal group samples clustered tighter together (range: 35–101 ng/mL), whereas, the overweight (range: 22–149 ng/mL) and obese (range: 18–143 ng/mL) had a considerably larger variable range, suggesting that weight may play a role in influencing levels of 2-AG in breast milk.

### 3.3. Diurnal and Longitudinal Changes in 2-AG Levels in Milk

**Figure 5:**
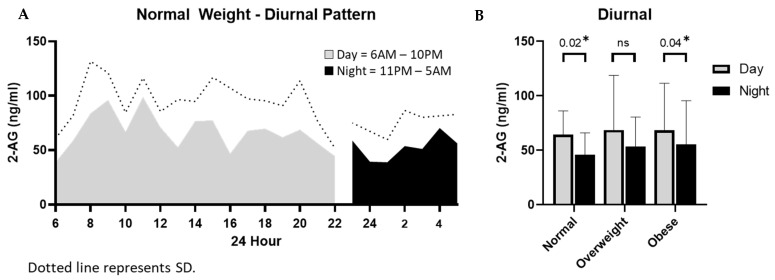
Effect of diurnal rhythm on breast milk endocannabinoid concentrations. (**A**). Milk samples were collected over a 24-h period and divided into day samples (6 am–10 pm) and night samples (11 pm–5 am) and analyzed for 2-AG concentrations. (**B**). Analysis was performed for each of the 3 BMI weight groups. Diurnal time points (day vs. night) for each weight group was analyzed using a paired Student’s *t* test with * *p* ≤ 0.05 being statistically significant.

Behavior and metabolic requirements may be influenced by circadian/diurnal rhythm. To determine the effect of diurnal rhythm on 2-AG in breast milk, samples were collected over a 24-h period and analyzed as day samples (6AM-10PM) or night samples (11PM-5AM). 2-AG levels in milk were significantly higher during the day as compared to night samples in both normal (*p* = 0.02) and obese (*p* = 0.04) groups (Figure 5).

**Figure 6:**
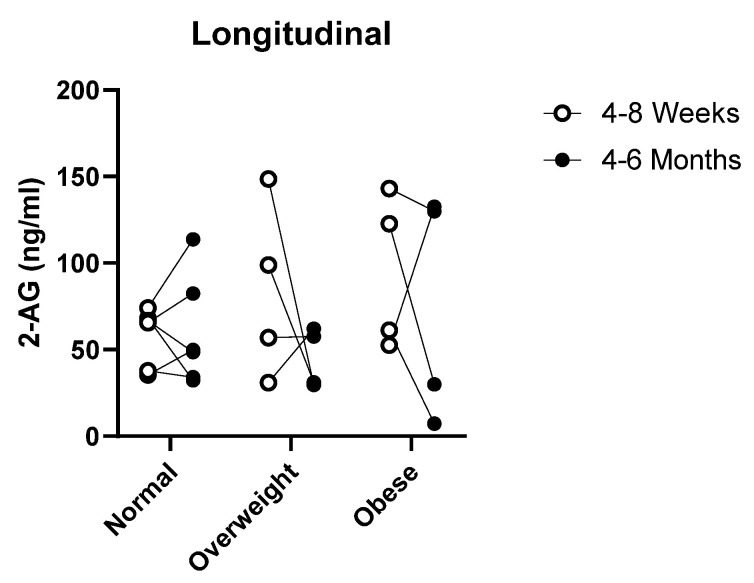
Breastfeeding longitudinal 2-AG concentrations. Normal (n = 6), overweight (n = 4) and obese (n = 4) donors provided samples between 4 and 8 weeks and again at 4-6 months for 2-AG analysis. ○ represents early time point between 4 and 8 weeks and ● represents a late time point between 4–6 months.

Longitudinal follow up was done 4–6 months postpartum after the initial collection of 4–6 weeks postpartum in order to determine any difference in the 2-AG levels overtime. Unfortunately, there was a high attrition rate due to the length of time between collection points for this part of the study, with only 14 out of 36 women contributing milk samples. No significant difference was observed for the average 2-AG levels overtime for the three groups (Figure 6). However, the average 2-AG concentrations in milk tended to decrease over time for the overweight and obese group, whereas the normal group remained consistent. 

## 4. Discussion

Endocannabinoids are bioactive lipid mediators critically involved in neonatal physiology. They were detected in both bovine and human milk as early as 1998 with 2-AG concentrations up to 1000-fold higher than AEA [16,18,19]. Unfortunately, the role and mechanisms of the action of endocannabinoids in breast milk and infant development are still poorly understood. An early study using an animal model demonstrated that infant feeding behavior centered on the activation of CB1 receptors and played a critical role in initiating milk suckling and in the growth and development during early stages of life [18,20]. Evaluating the direct role of the endocannabinoid system in human infant feeding is much needed and has become an increasing area of research. On review of the literature for endocannabinoid levels in human milk, we found various collection and storage methods before analysis which may account for the many discrepancies in reported endocannabinoid levels [16,21,22,23]. One case study with milk samples from a single donor at seven months of lactation demonstrated that milk storage introduced significant variability of endocannabinoid and oxylipins levels, especially storage at 4 °C [16].

The major importance of this study was to determine the normal baseline levels of AEA and 2-AG endocannabinoids in breast milk using strict collection and storage conditions, and also determine the effects of maternal obesity and long-term breastfeeding on their concentrations. Our initial experiment demonstrated that optimal processing and handling methods for milk samples is extremely important. In order to better understand the important biological implications of milk storage on endocannabinoid concentrations, we evaluated AEA and 2-AG levels at various storage temperatures for various durations, using quantitative UPLC MS/MS. The levels reported in samples stored at 4 °C increased over time and were significantly higher at 1 day (*p* = 0.01) or 1 week (*p* = 0.03). This may occur due to an increase in enzymatic activity which catalyzes the metabolic creation of 2-AG in milk, thus affecting endocannabinoid levels. Storage at −20 °C greatly reduced the endocannabinoid levels but were still significantly higher compared to fresh breast milk. There were virtually no changes in milk stored at −80 °C after one month. For this study, all milk samples were collected and stored under stringent conditions as described in the materials and methods.

Our study provides the first report describing endocannabinoid levels in human milk collected for a 24-h period at 4–8 weeks postpartum. AEA in breast milk has been reported to be very low and was below the limit of detection (<1.9 ng/mL) in our assay, whereas 2-AG (59.3 ± 18.3 ng/mL) levels were quite high for women with normal BMI. Gaitan et al. also previously reported very low concentrations of AEA (0.08 ± 0.01 ng/mL) as compared to 2-AG (312.11 ± 119.97 ng/mL) levels. [21] Together these data demonstrate that it is not a technical aspect in detecting AEA, but rather AEA is only present at extremely low levels. Interestingly, we also observed a larger but variable range of 2-AG in the overweight and obese groups. Obesity has been documented to alter endocannabinoid tissue and plasma levels of 2-AG [24]. At present, no single mechanism has been identified that explains the increased levels of endocannabinoids in obesity. It is also known that the more lipid-soluble a drug/molecule is, the higher the transfer into human milk. Therefore, we sought to determine if being overweight or obese may alter breast milk endocannabinoid levels. Although we did not find a significant difference between the average 2-AG levels for normal BMI compared to overweight and obese, the overweight and obese mothers exhibited a greater range in 2-AG levels compared to normal weight mothers which had a smaller and tighter clustered range. These data suggest that overweight mothers may have higher levels of 2-AG in their breast milk. However, with these small numbers, there was no significant correlation observed with an increase in weight and 2-AG levels. At this point, we cannot make any conclusions as to how this may affect the breastfed infant, but is a unique observation that needs to be explored.

Endocannabinoids exhibit a significant circadian rhythm in 2-AG concentrations in serum as reported by Hanlon et al. [25]. Breast milk also changes throughout the day and night with many nursing women noticing greater volume and faster flow in the early hours of the day. In other studies, breast milk produced during the day had three times higher levels of cortisol and at the end of the day contains more serotonin and other elements designed to help the baby sleep [26]. In our study, high levels of 2-AG were observed during the day. This trend was significant in both normal and obese groups. It is reasonable that milk 2-AG may also help support infants’ circadian rhythm. Hormones and immune elements passed into breast milk have been suggested to help infants define their own circadian rhythms during the early months of life.

Once lactation is established, the content of milk remains relatively constant for up to 6 months [27]. At present, little is known about the levels of endocannabinoid levels overtime. This led us to examine if the concentration of endocannabinoids changed overtime as well. Although the number of samples is limited due to a high attrition rate of donors in the study, we found some donors’ 2-AG levels increased and others decreased overtime with no statistical difference. However, we did note much varied changes in the levels of 2-AG at the 4–6-month time points for the overweight and obese group compared to the normal weight individuals. Although a variety of factors may influence endocannabinoid levels such as changes in stress, time of eating, exercising and sleep patterns, our data looking at BMI differences are interesting and suggest some correlation between 2-AG and maternal weight.

The strength of this study demonstrates the importance of appropriate collection, storage and analysis of breast milk in order to obtain accurate and reliable endocannabinoid measurements. Although we were unable to obtain a large number of milk samples for analysis as a function of maternal weight, we were able to see trends develop between 2-AG levels in milk and maternal weight.

## 5. Conclusions

Our study demonstrates that appropriate collection and storage of breast milk is critical for determining accurate endocannabinoid concentrations, and that storage at 4 °C for even one day significantly increases the level of 2-AG in the milk. The findings in this study also provide evidence that 2-AG could play a role in chrononutrition in communicating the time of day information for infants. In addition, we found mother’s weight post-partum may influence 2-AG levels in their human milk. Future studies need to be developed to correlate 2-AG in milk and outcome in their infants, specifically how increases in endocannabinoids in milk during storage may influence shaping the gut microbiota in early life. 

## Figures and Tables

**Table 1:** Demographics for Maternal–Infant Characteristics.

Characteristics	Mean ± SD or % (Frequency) or Range			
Maternal	Normal	Overweight	Obese	*p*-Value
	(N = 12)	(N = 11)	(N = 13)	
Age (yrs)	29.1 ± 5	30.6 ± 1.2	32 ± 4	0.238
Race (% (n/N))				0.030 *
White (non-hispanic)	50 (6/12)	90.09 (10/11)	92.3 (12/13)	
Hispanic	50 (6/12)	9.09 (1/11)	7.6 (1/13)	
BMI (kg/m^2^) (Mean ± SD)	22.4 ± 1.6	27.0 ± 1.2	36.8 ± 6.2	0.000 **
Post Pregnancy (at the time of study)				
Total number of births (% (n/N))				0.007 **
Primiparous	58.3 (7/12)	9.1 (1/11)	7.6 (1/13)	
Multiparous	41.7 (5/12)	90.9 (10/11)	46.1 (12/13)	
Previous breastfeeding experience (% (n/N))				0.007 **
Yes	33.3 (5/12)	90.9 (10/11)	92.3 (12/13)	
No	58.3 (7/12)	9.1 (1/11)	7.7 (1/13)	
Primary mode of feeding (% (n/N))				0.603
Breast only	66.6 (8/12)	81.8 (9/11)	84.6 (11/13)	
Breast and Pump	33.4 (4/12)	18.2 (2/11)	15.4 (2/13)	
Exercise				
Yes (% (n/N))	41.6 (5/12)	36.3 (4/11)	38.4 (5/13)	1.000
Times/week	3–7	3–4	2–3	
Hours/week	1–3	2–5	1–5	
Infant				
Sex (% (n/N))				0.839
Male	58.3 (7/12)	45.4 (5/11)	46.3 (6/13)	
Female	41.6 (5/12)	54.5 (6/11)	53.8 (7/13)	
Weight (lbs) (Mean ± SD)				
At birth	6.9 ± 0.7	7.6 ± 1	8.1 ± 1.2	0.019 *
At 4–8 weeks	9.6 ± 1.5	10.2 ± 1.6	9.9 ± 1.8	0.697
Feeds per 24 h	9 ± 1.6	9.2 ± 2.3	9.8 ± 1.8	0.550

## Data Availability

No data has been uploaded to any databases.
